# Under-diagnosis of Takotsubo Cardiomyopathy Increases Risk of Adverse Events: A Case Study

**DOI:** 10.7759/cureus.5749

**Published:** 2019-09-24

**Authors:** Harvinder S Power

**Affiliations:** 1 Imperial College London, Imperial College Nhs Trust, London, GBR

**Keywords:** takosubo, cardiomyopathy

## Abstract

Takotsubo cardiomyopathy (TC), or broken-heart syndrome, is characterized by high levels of adrenaline and noradrenaline leading to coronary artery vasospasm. Often presenting similarly to myocardial infarction (MI), Takotsubo is under-diagnosed in the population, typically leading to management under MI guidelines.

An 80-year-old woman presented with chest pain and electrocardiogram (EKG or ECG) changes but on further investigations, it emerged that the underlying pathology was not consistent with MI. Here, we highlight the importance of considering alternative diagnoses even with an initially seemingly clear-cut picture of MI. Due to the nature of the pathology, management of Takotsubo under MI protocols can lead to worsened outcomes. It is therefore of importance to increasingly consider TC as a diagnosis in cases of unclear MI.

## Introduction

In this case report, a recent case of Takotsubo cardiomyopathy (TC) is reviewed from start to finish, with all aspects of the history, examination, and relevant investigations. In this case, the diagnosis and management were delivered under the myocardial infarction (MI) protocol; however, management under MI protocols in TC can deteriorate patient outcomes. This report is aimed to highlight key factors in a presentation which may lead a clinician to diagnose TC instead of MI.

## Case presentation

An 80-year-old woman presented after a referral from her GP with chest pain and vomiting after mild exertion. Chest pain was reported to attenuate with rest after 30 minutes. Approximately three cups worth of vomit was produced, some redness in coloration, but was attributed to red wine consumed at lunch. Her chest pain was of band distribution around the chest, came on after vomiting, was six out of ten in severity and settled after resting for a few hours. Relevant negatives included no shortness of breath, calf-swelling, altered sense of taste, or change to exercise tolerance. Of note, the patient reported a similar episode of pain three months prior, with the family practitioner concluding this was musculoskeletal pain. Her past medical history was significant for rheumatic fever aged six, and an un-repaired hiatus hernia. She was on no regular medications, with no known drug allergies. Her family history was significant for diabetes mellitus on the paternal side, and ischemic stroke on the maternal side. Generally, she maintains activities of daily living well, with a normal walking tolerance of over two miles. On examination, there was a collapsing pulse, with no other significant findings.

Based on this initial history and presentation the following differential diagnoses were considered. Firstly, MI due to chest pain and vomiting present in history, rendering it vital to exclude. Stable angina due to chest pain on exertion, which was relieved after rest. Pulmonary embolism due to sudden onset of chest pain, however, no association of shortness of breath. Rheumatic heart fever due to the past medical history of rheumatic fever as a child. Rheumatic heart fever was mentioned in her history and was an important background for the cardiovascular examination. Collections of granulomatous tissue can lead to a build-up of fibrotic connective tissue on the heart valves.

**Figure 1:**
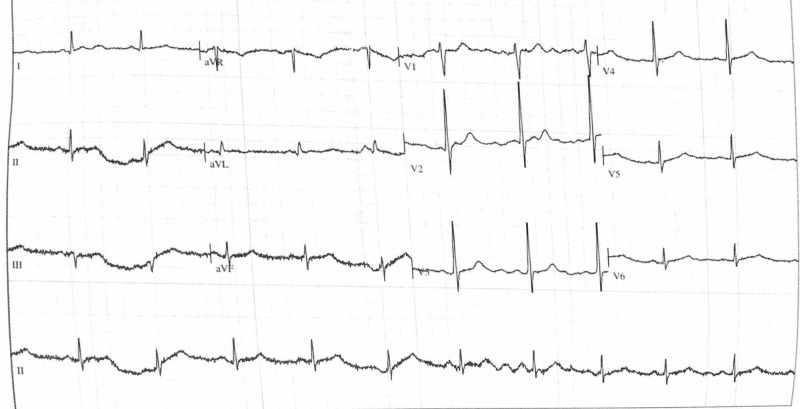
Electrocardiogram on admission. Mild regional ST-segment changes present, most noticeable in V2.

Her blood results showed a Troponin T (968 ng/L). All other blood results were within normal ranges. Electrocardiogram (EKG or ECG) showed ST-segment elevation in leads V3 and V4 with no further identifiable pathology. Figure 1 shows the full ECG on admission.

**Figure 2:**
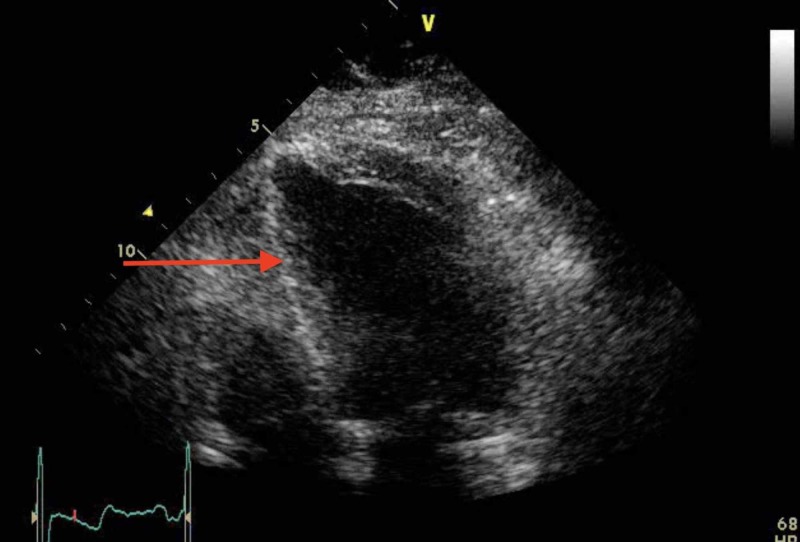
Echocardiogram on admission. Image taken during ventricular contraction. The red arrow highlights akinesia of the inferior segment with a severely akinetic apex.

Her echocardiogram was performed after her initial management was completed. Figure 2 shows an image of the echocardiogram on admission. It showed akinesia of inferior, posterior, and infero-lateral segments with a severely akinetic apex. Left-ventricular ejection fraction (LVEF) was 25%, with aortic regurgitation present. Her coronary angiography was performed after her initial management was completed and demonstrated normal arteries throughout with some mild plaques.

Based on the presentation, our patient was started on aspirin, fondaparinux, omeprazole, ticagrelor, bisoprolol, isosorbide mononitrate, atorvastatin, ramipril, and spironolactone.

Medical management was decided as per hospital trust guidelines which stratifies patients based on GRACE score, with our patient being deemed high risk (calculated six-month mortality risk of 24%). Our patient also reported having symptoms of heart failure (in particular, peripheral edema) shortly after admission which was the rationale behind the use of spironolactone. The patient was stable and discharged before a definitive diagnosis was made, with a follow up in an outpatient clinic.

Definitive diagnosis

After the results of the coronary angiogram showed normal arteries throughout, our differentials changed significantly. Whilst the ECG indicated STEMI, the results of the coronary angiogram did not align with this. On consideration, transient coronary ischemia would lead to ECG changes, however, not enough plaques seen for this to be likely. Pericarditis could explain a clear coronary angiography, but the absence of ECG changes indicates otherwise. Syndrome X was a potential differential due to angina symptoms with normal coronary vasculature, however, unlikely given the repeat echocardiogram. On balance, the most likely diagnosis given all evidence was TC.

**Figure 3:**
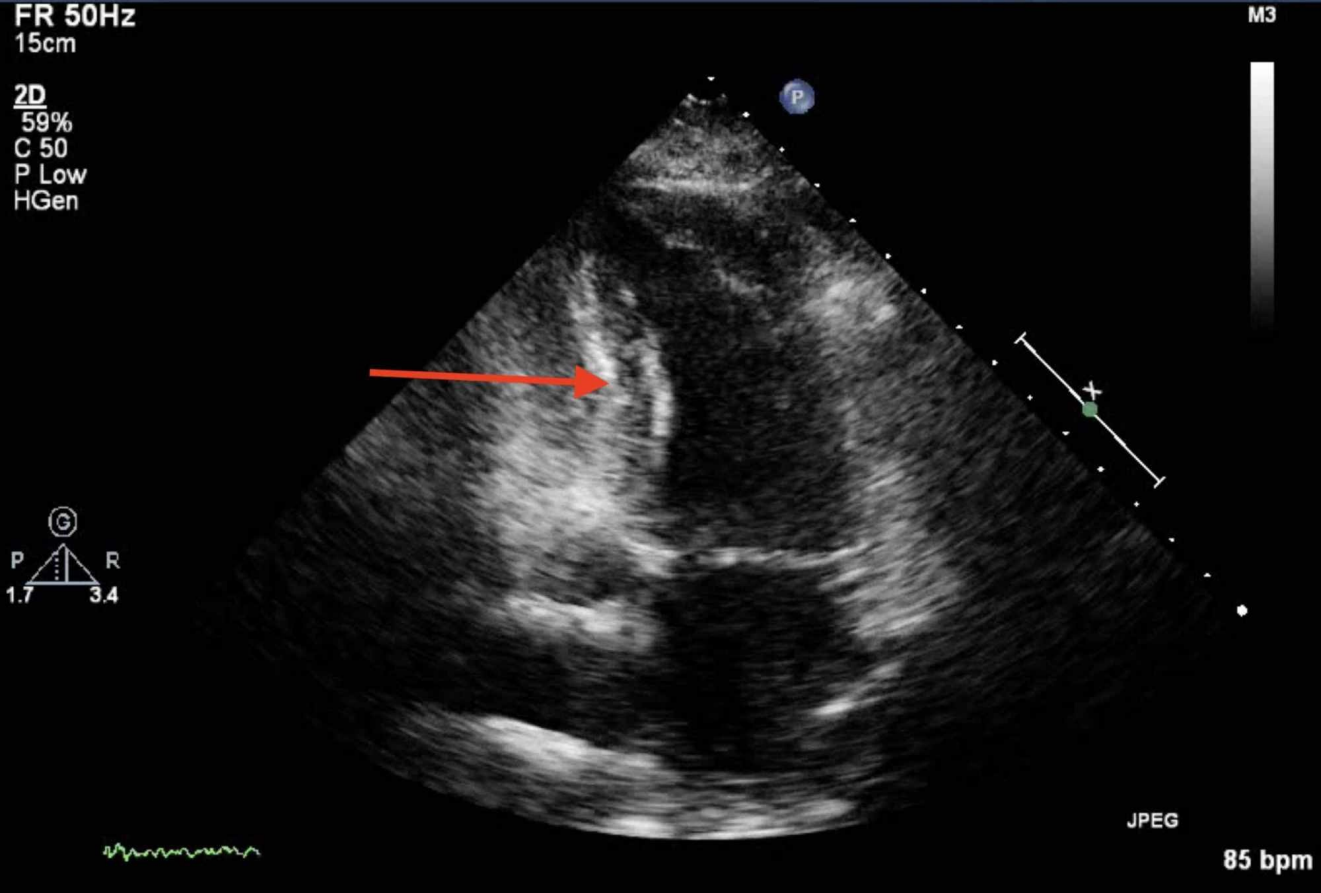
Echocardiogram at follow-up showing improvement of akinesia compared to echocardiogram on admission. Image taken during ventricular contraction. Red arrow demonstrates the area of improved wall motion.

Our patient was followed up in an outpatient clinic to assess her response. At follow-up, our patient's repeat echocardiogram demonstrated significant improvement, with a left ventricular ejection fraction of 64% (improved from 25% on admission). Figure 3 shows an image of the echocardiogram at follow-up. The seemingly transient nature of the reduced cardiac function solidifies the diagnosis of TC in contrast to other differentials.

Also known as stress cardiomyopathy, or broken-heart syndrome, TC is a condition where there is a transient weakening of the heart muscle, often triggered by emotional stress.

## Discussion

Based on the history alone, it seemed to be a clear diagnosis of angina. However, with the addition of further information, the differentials changed significantly. This highlights the importance of physical examination, and investigations, in particular, linking them all together rather than taking one in isolation.

Patients with TC often present with chest pain and an ECG which mimics anterior wall MI. Epidemiologically, the condition is found in approximately 1%-2% of patients presenting with acute coronary syndrome, and primarily affects postmenopausal women for reasons which are unknown [1]. The condition is poorly understood, but it is well documented that there is an association with emotional or physical stress.

This highlights the pertinence of the social history - it may have been found that there was a recent family event which was causing significant stress, such as to precipitate the presentation of TC. Hence, it is imperative to sensitively ask patients if there has been any recent change to their social situation or any stress which they are under, as it has been well documented that stress plays a role in a considerable number of pathologies, including TC.

The mechanism of TC is known to involve catecholamines which can lead to either a stunned myocardium, directly mediated myocardial toxicity, microvascular dysfunction, or a multi-vessel coronary artery spasm [1]. The exact nature of TC is yet to be determined [2]. Drugs such as epinephrine and dobutamine are well understood to trigger TC due to increased circulating levels of catecholamines, with sympathomimetics such as midodrine and ergotamine tartrate also having reported cases of TC [3].

Interestingly, evidence has found that TC may be a protective mechanism by the body. In their research, it was found that excessively high levels of adrenaline in mice led to a reduction in pumping power through alteration of the activation pathway, protecting against toxic doses of adrenaline [4]. Some reports indicate that a genetic predisposition may exist, associated with the FMR1 gene [5].

Patients with TC typically show a more modest rise in creatine kinase-MB and cardiac troponin when compared to MI [6]. Coronary angiography typically shows normal coronary arteries, with 15% of patients showing obstructed coronary arteries [7].

The gold standard for diagnosis of TC is to use radio-contrast dye with a ventricular flow study to assess contraction of the ventricle [8]. In TC, the left ventricle fails to contract at the bottom (akinesia), with normal contraction towards the atrial end of the ventricle.

From a review of the literature, it becomes clear that TC is under-diagnosed in many cases. Work by Otten et al. highlights this, and advocates that many patients are not diagnosed with TC due to lack of knowledge amongst the scientific community about the condition [9]. Work is required to inform more health care professionals about this condition.

There is no definitive treatment for TC, with treatment being generally conservative [10]. Whilst patients may often have low blood pressures, inotropes such as digoxin and amiodarone can have a detrimental effect on outcomes due to high levels of catecholamines from the precipitating emotional or physical stress [10]. Medications containing nitrates are not recommended in TC. Research from the Australian Heart Research foundation has found that patients often have enhanced nitric oxide signaling [11]. In this case study, a nitrate was prescribed, fortunately, it was reported to have no adverse outcome. Previous case reports have mentioned the negative consequences of inotrope usage, in particular, hemodynamic compromise [12]. The main recommended therapy is conservative avoidance of stress, with regular exercise [10].

## Conclusions

From this case there are a number of lessons to be learnt. A holistic approach to healthcare is of vital importance, and emphasis needs to be placed on the social history to determine any stressors. Additionally, physicians should always consider alternative diagnoses when presented with a seemingly clear-cut presentation. Whilst inotropes did not have an adverse outcome in this case, it could have harmful effects in other cases, preventable through a lateral approach to diagnostic formulation.
